# Influenza Classification Suite: An automated Galaxy workflow for rapid influenza sequence analysis

**DOI:** 10.1111/irv.12722

**Published:** 2020-02-16

**Authors:** Diane Eisler, Dan Fornika, Lauren C. Tindale, Tracy Chan, Suzana Sabaiduc, Rebecca Hickman, Catharine Chambers, Mel Krajden, Danuta M. Skowronski, Agatha Jassem, William Hsiao

**Affiliations:** ^1^ British Columbia Centre for Disease Control Provincial Health Services Authority Vancouver BC Canada; ^2^ University of British Columbia Vancouver BC Canada

**Keywords:** data analysis, Galaxy, genomics, Influenza, workflow

## Abstract

Influenza viruses continually evolve to evade population immunity, and the different lineages are assigned into clades based on shared mutations. We have developed a publicly available computational workflow, the Influenza Classification Suite, for rapid clade mapping of sequenced influenza viruses. This suite provides a user‐friendly workflow implemented in Galaxy to automate clade calling and antigenic site extraction. Workflow input includes clade definition and amino acid index array files, which can be customized to identify any clades of interest. The Influenza Classification Suite provides rapid, high‐resolution understanding of circulating influenza strain evolution to inform influenza vaccine effectiveness and the need for potential vaccine reformulation.

## 1 INTRODUCTION

Influenza viruses evolve continually, necessitating frequent vaccine reformulation to effectively protect the population.^1^ To monitor circulating variants and their impact on vaccine effectiveness (VE), networks such as the Canadian Sentinel Practitioner Surveillance Network (SPSN) sequence the hemagglutinin (HA) gene of influenza‐positive patient samples. In addition to VE, enhanced routine influenza surveillance could lead to a better understanding of virus evolution, reassortment, and antiviral resistance, which could help improve early warning systems and pandemic preparedness.

Influenza clade calling is typically done using in‐house scripts based on amino acid (AA) clade‐defining substitutions and often paired with phylogenetic analyses to account for mismatched or missing AA among the clade‐defining AAs. Generating these clade definitions remains the greatest challenge, requiring careful and frequent monitoring of global influenza surveillance reports and sequence data. Nextstrain, a processing pipeline and browser‐based visualization tool for sequence data, is a powerful tool for influenza phylogenetic analysis because it has pre‐established clade definitions that are updated as required.^2^ A barrier to routine use is the need to install and update the Nextstrain build as clade definitions change or to upload user data to the Global Initiative for Sharing All Influenza Data's (GISAID)^3^ FluSurver App to use a current version of Nextstrain. Furthermore, clade definitions cannot be changed to highlight emerging clade subgroups. Another challenge in clade calling is that parallel evolution, identical substitutions arising in different clades, can confuse phylogeny algorithms into grouping distinct clades based on parallel mutations.^4^ Other pipelines such as octoFLU, originally developed for swine influenza strains in North America, classify lineages and clades based on phylogenetic analysis alone using an extensively curated reference gene data set.^5^ In the context of rapidly evolving seasonal human influenza, this reference data set would require constant updating.

To modernize the labor‐intensive approaches previously involved in assigning clade designation and interpreting antigenic site relatedness between circulating viruses and the vaccine strain and address limitations of using currently available pipelines, we have developed an “Influenza Classification Suite,” an automated Python‐based workflow, publicly available through the popular Web‐based platform Galaxy that is used for the development and distribution of bioinformatic pipelines.^6^ Automated and documented workflows are critical for generating reproducible results^7^ and for increasing near real‐time availability of virological surveillance information. The Influenza Classification Suite can be easily implemented and modified by influenza researchers to automate the process of clade calling in their analysis pipelines. We describe our workflow and demonstrate its application to HA gene sequences using a test data set from influenza A(H3N2)‐positive specimens collected by SPSN sites across Canada during the 2016/17 epidemic.^8^

## 2 IMPLEMENTATION

**Figure 1:**
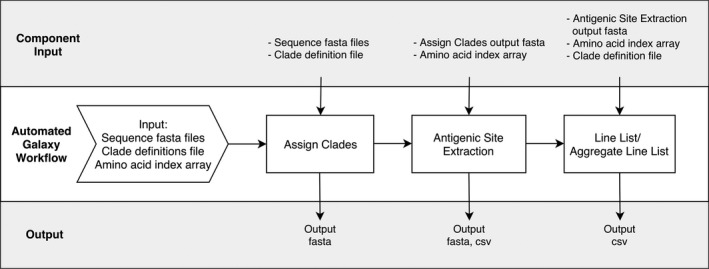
Flowchart illustrating the Influenza Classification Suite in Galaxy. We have created an automated workflow as shown in the center diagram, although all tools can also be run independently

Sanger sequencing of hemagglutinin (HA) genes is routinely performed by SPSN researchers for influenza‐positive specimens collected from patients presenting with an influenza‐like illness. We created Python scripts to automate the labor‐intensive manual clade calling and antigenic site identification process, and validated output from each script against manually obtained results. Each script is defined as a stand‐alone tool in Galaxy (Table 1) and is also combined into a standardized analysis workflow. All tools can also be run in command line for integration into existing pipelines. The source code of the tools is publicly available on GitHub (https://github.com/Public-Health-Bioinformatics/flu_classification_suite), with the option to install the tools easily using the Conda package manager. A workflow was created by combining tools in a pipeline to automate a series of tasks in a standardized, user‐friendly manner (Figure 1). Our comprehensive user's manual, template files, and test data can be found in our GitHub README.md.

**Table 1:** Description of Galaxy tools in the Influenza Classification Suite

Galaxy tool name	Workflow description
Assign Clades	Uses a clade definition file to assign and append clade designations to sequence names in influenza FASTA files.
Antigenic Site Extraction	Uses an influenza subtype‐specific amino acid index array to extract antigenic amino acids from influenza sequences and output to FASTA.
Line List	Transforms FASTA files of influenza antigenic maps into line lists.
Aggregate Line List	Transforms FASTA files of influenza antigenic maps into line lists, summarizing occurrences of each sequevar.
Change FASTA Deflines	Changes sequence names in a FASTA file, according to old and new names specified in a text (.csv ortxt) file.

## 3 RESULTS

### 3.1 Tool: Change FASTA Deflines

“Change FASTA Deflines” was included as a tool to automate changing or de‐identifying sequence names in FASTA files. It uses a two‐column text file (comma‐ or tab‐separated) containing existing sequence names in column 1 and desired sequence names in column 2. The program searches the target FASTA file for definition lines matching those in column 1 and, if found, changes them as specified in column 2, allowing fast and accurate renaming.

### 3.2 Tool: Assign Clades

**Figure 2:**
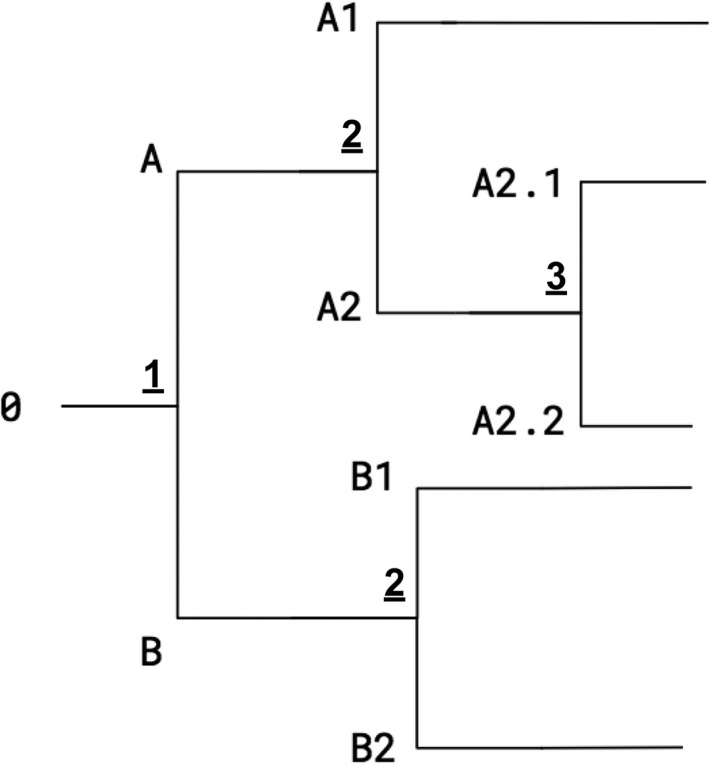
Cladogram demonstrating how child clades evolve from parent clades over time. Underlined bolded numbers represent the depth parameter as specified in the clade definition file

A clade definition file is used to assign and append clade designations to influenza sequence files. Viral clades are inherently nested, with child clades evolving from parent clades over time (Figure 2). To determine whether sequences matched a clade, we required clade definitions to (a) contain the clade name(s), (b) contain respective clade‐defining AA and position numbers, and (c) be easily modified with common software. An Excel template is provided in our GitHub repository that allows researchers to easily edit and define clades in comma‐separated value (CSV) format (Table 2). We further incorporated a “depth” parameter into each clade definition to resolve situations in which a sequence is an exact match to more than one clade (eg, parent and child clade). The depth parameter is an integer greater than 0, defining the relative ancestry of the clades (eg, parent clade depth = 2 and child clade depth = 3). The European Centre for Disease Prevention and Control (ECDC) Influenza Virus Characterization Reports (https://www.ecdc.europa.eu) and Nextstrain can be used in identifying and naming clade‐defining substitutions.^2^

**Table 2:** Example of clade definition file format (.csv). A template is available to use and edit in our GitHub repository

Clade Name	Depth^b^	AA^a^ Position 1	AA Identity 1	…	AA Position N	AA Identity N
A	1	3	I	…	171	K
B	1	3	I	…	171	K
A1	2	3	I	…	225	D
A2	2	3	I	…	160	T
B1	2	3	I	…	160	T
B2	2	3	I	…	188	M
A2.1	3	3	I	…	171	A
A2.2	3	24	K	…	188	R

a AA, amino acid.

b Depth is an integer greater than 0, defining the relative ancestry of the clades (eg, parent clade depth = 2 and child clade depth = 3).

To assign clades to sequence results, the Galaxy tool “Assign Clades” reads in the clade definition file (CSV) and the aligned AA sequence files (FASTA). In the clade definition file, each clade is represented by a tuple (an ordered list) consisting of a clade name, depth, and a Python dictionary of AA positions mapping to AA identities. The full‐length AA FASTA sequences are read into a list of Biopython SeqRecords, and each SeqRecord is compared to each clade definition. If the list of matches contains more than one clade, depth parameters of all matching clades are compared, and the sequence is assigned the clade with the highest depth. The SeqRecord is renamed by appending an underscore, followed by the clade name (or “No_match” if no clades were an exact match), and all sequences are output to FASTA format with their clade designations.

### 3.3 Tool: Antigenic Site Extraction

Since antigenic sites of influenza A/H1 and A/H3 subtypes and B/Victoria and B/Yamagata lineages comprise different AA positions,^9^, ^10^, ^11^ these are specified to programmatically extract from the full‐length HA sequence. This was done using CSV files containing an array of respective antigenic site positions; files are available in our GitHub repository.

To extract antigenic AAs from sequences, the Galaxy tool “Antigenic Site Extraction" reads in the AA index array (CSV) and the “Assign Clades” output file (FASTA). The AA index array is the list of subtype/lineage‐specific indices to extract from the sequence FASTA files. The full‐length FASTA sequences, containing clade names, are read into SeqRecords, and the specified antigenic AAs from original sequences are extracted and the antigenic sites are written to FASTA or CSV format with clade‐defined sequence names.

### 3.4 Tool: Line List

The Galaxy tool “Line List” reads in FASTA files of a reference strain (eg, vaccine strain) and the antigenic sites output from the previous step as SeqRecords. “Line List” compares AAs of each sample sequence to the corresponding reference strain AA and replaces identical AAs with dots (ie, “.”) for display purposes. It outputs the following to CSV format, for import into spreadsheet software: (a) column headers, (b) antigenic map position numbers (from AA index array), (c) reference strain AA sequence, and (d) sequence names, clade names, and antigenic map sequences. This custom format allows for easy determination of which clades (subclades and/or subgroups) are present, how these compare to the vaccine strain, the sites at which they differ, and the percent identity of each sequence to the reference strain and the number of substitutions. The Galaxy tool “Aggregate Line List” is used to aggregate identical antigenic maps when the number of sequences is too great to view in the allowed space. It performs all of the functions as “Line List” and collapses identical sequences within clades, enumerating them and displaying the count in a separate column.

### 3.5 Downstream application

The output FASTA files can be used for downstream phylogenetic analysis and constructing dendrograms using a variety of programs.

### 3.6 Influenza Classification Suite validation

Output from each programmatic analysis step was compared using a test set of 574 clinical influenza A(H3N2)‐positive patient sequences from the SPSN 2016/17 HA data set and corresponding manually obtained results.^8^, ^12^ With a stringent clade assignment cutoff of 100% sequence match to a given clade, 557/574 sequences (97%) matched. Of the 17 “No_Match” sequences (3%), 10 had incomplete sequences at a specific AA position used in the clade definition and seven had a mutation in a clade‐defining AA. “No_Match” sequences were analyzed individually, and clades were manually assigned. Insertions/deletions (indels) are reflected in the aligned FASTA input files, and frequent indels are accounted for when designing the clade definition files. Rare one‐off indels are not captured by the software and will also be flagged as “No_Match” for manual analysis. Using the Influenza Classification Suite Galaxy pipeline, manual analysis time was reduced by hours, with increased reliability and reproducibility. The pipeline is now in routine use by the SPSN sequencing team.

## 4 CONCLUSIONS

This publicly available Influenza Classification Suite provides an automated and reproducible pipeline that can be used to greatly increase the ease and efficiency of HA analysis. The pipeline is also fully customizable to respond to emerging variants within any influenza subtype or gene segment by selecting the respective Excel‐modifiable clade definition file, FASTA input file, and AA index array—for example, the pipeline could be adapted to identify antigenic sites in influenza B sequences, or antiviral resistance AA positions in NA sequences. Manual analysis that required several programs and hours to complete can now be analyzed in minutes using this Influenza Classification Suite.

Compared to implementing the octoFLU^5^ pipeline with in‐house human influenza curated reference gene data sets, the clade definition file and AA index array of antigenic sites required as input by the Influenza Classification Suite are easier to curate from lists upkept by the ECDC and Nextstrain.^2^ The Influenza Classification Suite also gives influenza researchers the ability to investigate emerging clade subgroups before they are officially updated by Nextstrain. While Nextstrain corrects for parallel evolution errors by masking parallel sites, in our Suite the depth levels assigned to each clade in the clade definition file can be optimized for correct clade calling.

Rapid and more automated processing of influenza HA sequences is important for real‐time tracking of influenza strain evolution. This information is important to understand emerging circulating influenza escape variants, their relation to vaccine‐virus relatedness, and helps guide vaccine strain reformulation.

## References

[1] Petrova VN , Russell CA . The evolution of seasonal influenza viruses. Nat Rev Microbiol. 2018;16(1):47‐60. 29081496 10.1038/nrmicro.2017.118

[2] Hadfield J , Megill C , Bell SM , et al. Nextstrain: real‐time tracking of pathogen evolution. Kelso J, ed. Bioinformatics. 2018;34(23):4121‐4123. 29790939 10.1093/bioinformatics/bty407 PMC6247931

[3] Shu Y , McCauley J . GISAID: Global initiative on sharing all influenza data – from vision to reality. Eurosurveillance. 2017;22(13):30494. 28382917 10.2807/1560-7917.ES.2017.22.13.30494 PMC5388101

[4] Bedford T , Huddleston J , Potter B , Neher RA . Seasonal influenza circulation patterns and projections for September 2019 to September 2020. bioRxiv. 2019 10.1101/780627 [Epub ahead of print].

[5] Chang J , Anderson TK , Zeller MA , Gauger PC , Vincent AL . octoFLU: Automated Classification for the Evolutionary Origin of Influenza A Virus Gene Sequences Detected in U.S. Swine. Microbiol Resour Announc. 2019;8(32):e00673. 31395641 10.1128/MRA.00673-19 PMC6687928

[6] Afgan E , Baker D , Batut B , et al. The Galaxy platform for accessible, reproducible and collaborative biomedical analyses: 2018 update. Nucleic Acids Res. 2018;2018(46):537‐544. 10.1093/nar/gky379 PMC6030816 29790989

[7] Sandve GK , Nekrutenko A , Taylor J , Hovig E . Ten simple rules for reproducible computational research. PLoS Comput Biol. 2013;9(10):e1003285. 24204232 10.1371/journal.pcbi.1003285 PMC3812051

[8] Skowronski DM , Chambers C , Sabaiduc S , et al. Interim estimates of 2016/17 vaccine effectiveness against influenza A(H3N2), Canada, January 2017. Eurosurveillance. 2017;22(6):1‐8. 10.2807/1560-7917.ES.2017.22.6.30460 PMC5316907 28205503

[9] Ndifon W , Wingreen NS , Levin SA . Differential neutralization efficiency of hemagglutinin epitopes, antibody interference, and the design of influenza vaccines. Proc Natl Acad Sci USA. 2009;106(21):8701‐8706. 19439657 10.1073/pnas.0903427106 PMC2688967

[10] Brownlee GG , Fodor E . The predicted antigenicity of the haemagglutinin of the 1918 Spanish influenza pandemic suggests an avian origin. Philos Trans R Soc Lond B Biol Sci. 2001;356(1416):1871‐1876 *.* 11779386 10.1098/rstb.2001.1001 PMC1088563

[11] Wang Q , Cheng F , Lu M , Tian X , Ma J . Crystal structure of unliganded influenza B virus hemagglutinin. J Virol. 2008;82(6):3011‐3020. 18184701 10.1128/JVI.02477-07 PMC2259021

[12] Skowronski DM , De Serres G . Role of egg‐adaptation mutations in low influenza A(H3N2) vaccine effectiveness during the 2012–2013 season. Clin Infect Dis. 2018;67(9):1474‐1476. 29688295 10.1093/cid/ciy350 PMC6186855

